# Determining the impact of a new physiotherapist-led primary care model for back pain: protocol for a pilot cluster randomized controlled trial

**DOI:** 10.1186/s13063-017-2279-7

**Published:** 2017-11-09

**Authors:** Jordan Miller, David Barber, Catherine Donnelly, Simon French, Michael Green, Jonathan Hill, Joy MacDermid, Jacquelyn Marsh, Kathleen Norman, Julie Richardson, Monica Taljaard, Timothy Wideman, Lynn Cooper, Colleen McPhee

**Affiliations:** 1 School of Rehabilitation Therapy, Queen’s University, 31 George Street, Kingston, Ontario K7L 3N6 Canada; 2 Department of Family Medicine, Queen’s University, Kingston, Canada; 3 Keele University, Keele, UK; 4 Physical Therapy, Western University, London, Canada; 5 School of Rehabilitation Science, McMaster University, Hamilton, Canada; 6 Ottawa Hospital Research Institute, Ottawa, Canada; 7 School of Physical and Occupational Therapy, McGill University, Montreal, Canada; 8 Canadian Pain Coalition, Oshawa, Canada; 9 McMaster Family Health Team, Hamilton, Canada

**Keywords:** Low back pain, Primary care, Physiotherapy, Cluster randomized trial

## Abstract

**Background:**

Back pain is a leading contributor to disability, healthcare costs, and lost work. Family physicians are the most common first point of contact in the healthcare system for people with back pain, but physiotherapists (PTs) may be able to support the primary care team through evidence-based primary care. A cluster randomized trial is needed to determine the clinical, health system, and societal impact of a primary care model that integrates physiotherapists at the first visit for people with back pain. Prior to conducting a future fully powered cluster randomized trial, we need to demonstrate feasibility of the methods. Therefore, the purpose of this pilot study will be to:

1. Determine feasibility of patient recruitment, assessment procedures, and retention.
2. Determine the feasibility of training and implementation of a new PT-led primary care model for low back pain (LBP)
3. Explore the perspectives of patients and healthcare providers (HCPs) related to their experiences and attitudes towards the new service delivery model, barriers/facilitators to implementation, perceived satisfaction, perceived value, and impact on clinic processes and patient outcomes.

**Methods:**

This pilot cluster randomized controlled trial will enroll four sites and randomize them to implement a new PT-led primary care model for back pain or a usual physician-led primary care model. All adults booking a primary care visit for back pain will be invited to participate. Feasibility outcomes will include: recruitment and retention rates, completeness of assessment data, PT training participation and confidence after training, and PT treatment fidelity. Secondary outcomes will include the clinical, health system, cost, and process outcomes planned for the future fully powered cluster trial. Results will be analyzed and reported descriptively and qualitatively. To explore perspectives of both HCPs and patients, we will conduct semi-structured qualitative interviews with patients and focus groups with HCPs from participants in the PT-led primary care sites.

**Discussion:**

If this pilot demonstrates feasibility, a fully powered trial will provide evidence that has the potential to transform primary care for back pain. The full trial will inform future service design, whether these models should be more widely implemented, and training agendas.

**Trial registration:**

ClinicalTrials.gov, NCT03320148. Submitted for registration on 17 September 2017.

**Electronic supplementary material:**

The online version of this article (doi:10.1186/s13063-017-2279-7) contains supplementary material, which is available to authorized users.

## Background

Back pain is the leading cause of years lived with disability [1]; it costs the Canadian healthcare system between $6 and $12 billion annually [2], and is a leading contributor to lost work productivity [3, 4]. The burden of back pain on the Canadian healthcare system is further evidenced by frequent healthcare utilization. The Canadian Institute for Health Information (CIHI) suggests that low back pain (LBP) was the sixth most common reason for emergency department visits in 2010–2011, with over 150,000 visits [5]. Back pain is also associated with a large number of costly and unnecessary specialist consultations and diagnostic procedures [4, 6].

Back pain is the fifth most common reason for visiting a primary care physician [7–9]. While physicians are the most common first point of contact in the healthcare system for people with back pain, questions remain whether better patient outcomes could be achieved by involving other healthcare providers (HCPs) within the primary care team. For example, the use of physiotherapists (PTs) at the first visit might assist in the delivery of evidence-based treatments, and greater use of pharmacists might help prevent current increases in opioid prescription. Hartvigsen et al. [10, 11] argue for new primary care models to support physicians based on physicians only receiving a few hours of musculoskeletal training [12, 13] and low confidence in managing back pain [14, 15]. Including PTs in the primary care team is one way of helping address the workload challenge. If more patients with back pain are given the choice of seeing a PT first, it is hypothesized that this could ease the workload on physicians, provide a more focused back pain consultation, and improve outcomes.

Several governments and health organizations have identified team-based primary care as an important strategy to improve the effectiveness and sustainability of healthcare systems [16]. Advantages include improved access to appropriate care, care coordination, and self-management support. PTs may be an important team member to integrate into primary care for people with back pain. Evidence suggests that guideline-adherent care for back pain improves function and disability [17, 18], and that PTs can effectively implement recommendations from primary care guidelines for back pain [19–22], including: screening for red flags and the need for diagnostic imaging [23–25]; screening for risk factors for poor recovery [26–28]; providing reassurance, advice about physical activity, and exercise recommendations [29]; and delivering targeted, psychologically informed interventions for those at greater risk of poor recovery [28]. This body of evidence suggests trained PTs could play a greater role in providing guideline-adherent primary care for people with back pain.

There is growing evidence to suggest that early referral to PTs for back pain can reduce healthcare costs and improve access to appropriate care. For example, a study in the United States by Fritz et al. (*n* = 32,070) demonstrated that early PT referral was associated with reduced diagnostic imaging (odds ratio (OR) = 0.34), physician visits (OR = 0.26), surgeries (OR = 0.45), and use of opioid medications (OR = 0.78) over a 1-year period [30]. The mean reduction per person in healthcare costs associated with early physiotherapy has been reported to be from US$1202 to US$2736 [30, 31]. Furthermore, PTs in advanced practice roles have demonstrated accurate assessment, appropriate screening, and guideline-consistent triage [32], while reducing spinal surgeon waiting times [33]. Research to date has evaluated a model of care that includes a primary care visit with the physician followed by early referral to a PT. The proposed research will build on this evidence by determining the feasibility of having PTs available to patients as the first point of contact in the primary care team for back pain.

There is emerging evidence from other settings that suggests that PTs can adopt the primary care role with good clinical outcomes and improvements in healthcare efficiency. Studies in the United States military have shown that when PTs provide the first point of contact care for those with work-related injuries this produces high satisfaction ratings, faster access, decreased sick calls, and more appropriate use of PTs and physicians [24, 34–38]. Evidence on the transition from physician referral to direct access physiotherapy in the United Kingdom National Health Service found higher levels of patient and physician satisfaction, shorter physician wait times, fewer work absences, fewer diagnostic images, and lower prescription medication use [39–41]. These results are promising, but more rigorous evaluation methods and evidence specific to back pain and the Canadian primary care context are needed.

While PTs can provide effective care for back pain, there are unique features of PTs practicing in primary care that make it critical to rigorously evaluate new models of care that incorporate PTs in primary care teams. First, the majority of primary care visits are for multiple health concerns [42]. It is important to evaluate how new models of care impact the management of the population most often seen in primary care, people with multimorbidity and complex healthcare needs. Second, PTs in Canada do not have independent prescribing rights. Several centers in Canada have developed medical directives that allow PTs to prescribe nonsteroidal anti-inflammatory drugs under the authority of a physician (e.g., for short-term pain relief in LBP [22, 43, 44]), but these models need further evaluation. Third, it is unclear whether patients or other HCPs are satisfied with this model. One Canadian survey suggested high levels of satisfaction and confidence in advanced practice PTs, but this study was based on only 1% response to an electronic survey [45]. It is essential to better understand the perspectives of patients and HCPs to inform a PT-led primary care model for back pain.

The overarching aim of this line of research is to evaluate a novel primary care model for back pain that incorporates a PT at the first point of contact within the primary care team for people with back pain. The planned fully powered cluster randomized trial will have the following objectives:

1. Determine the effectiveness of a PT-led primary care model for back pain on individual health outcomes (function, pain intensity, quality of life, global rating of change, and adverse events) in comparison to usual physician led primary care.
2. Determine the impact of a PT-led primary care model for back pain on the healthcare system and society (healthcare accessibility, healthcare utilization, and cost-effectiveness).

Prior to conducting the planned fully powered cluster randomized trial, we need to test and demonstrate the feasibility of the trial methods. To determine the feasibility of a fully powered cluster randomized trial, this protocol is for a pilot cluster randomized trial with the following objectives over a 1-year period:

1. Determine the feasibility of patient recruitment, assessment procedures, and retention.
2. Determine the feasibility of training and implementation of a new PT-led primary care model for back pain.
3. Explore the perspectives of patients and HCPs related to their experiences and attitudes towards the new service delivery model, barriers/facilitators to implementation, perceived satisfaction, perceived value, and impact on clinic processes and patient outcomes.

## Methods

### Design

This is a pilot cluster randomized controlled trial at four primary care sites, with two sites randomized to a new PT-led primary care model for back pain and two sites randomized to usual physician-led primary care. A cluster randomized trial was chosen because the intervention includes integrating the PT into the clinical team and clinic processes. This integration would make using a traditional randomized controlled trial design difficult and would introduce a high risk of contamination for the usual care group. See Additional file 1 for completed SPIRIT checklist. To explore perspectives of both HCPs and patients, we will also conduct qualitative interviews with patients and focus groups with HCPs who participated in the PT-led primary care intervention.

### Enrollment and randomization of sites

For the pilot trial, four primary care practices in Kingston, Ontario, Canada (Family Health Teams or Community Health Centers) with a minimum of 2500 registered patients and two physicians each will be enrolled. We will purposefully invite one community health center and three family health teams to determine feasibility of the protocol in both settings. Sites will be sorted in order of size (number of registered patients) and placed into two groups (the largest site and smallest site forming one group and the middle two sites forming the other group). The two groups will then be randomly allocated to intervention or control arms by a statistician independent of the enrollment of sites and collection of data using computer-generated random numbers. The statistician will be blinded to the site names using a concealed list of anonymized codes for each site. We decided to randomize using these two groups in order to balance the group sizes for this pilot study given the large differences in number of registered patients across anticipated sites (ranging from 3000 to 16,000 patients). We anticipate using a stratified block randomization based on the number of registered patients for the full trial.

### Blinding

HCPs and patients will not be blinded due to the nature of the interventions being compared; that is, it is difficult to blind patients or care providers to the models of care being compared. All participants will be informed that the trial is comparing a model of care that involves integrating a physiotherapist within the primary care team at the first point of contact with the team and a usual care model that involves seeing their own family physician or nurse practitioner first. Since the primary outcome measures are self-report measures completed by patients, the assessors will also not be blinded to their allocation.

### Patient enrollment

Medical secretaries will screen patients for willingness to be invited to participate when they call to book an appointment. Patients who agree to being invited to participate will be booked with an onsite research assistant 30 min before their appointment time. The research assistant will provide information about the study, obtain informed consent, and enroll consenting patients at the time of their initial visit. Enrollment will take place over a 14-week period starting in September 2017.

### Inclusion/exclusion of patients

All adult (18 years and over) patients who ask to book a primary care visit related to back pain of any duration at any of the four participating primary care sites will be invited to participate. The primary care visit may be a first or repeat visit. Only patients who do not consent or those who report not being able to understand, read, and write English will be excluded.

### Research ethics

This study has been approved by the Health Science and Affiliated Teaching Hospitals Research Ethics Board (HSREB #6021536). All participants provided informed consent for participation. The study letter of information and consent has been included in Additional file 2.

### Interventions

#### Physiotherapist-led primary care model for back pain

The index intervention will be incorporating a PT within the primary care team at the first point of contact for people with back pain at no cost to the patient. Patients in this model will be given the choice of seeing the PT or family doctor. They will be encouraged to book with the PT except when the primary reason for the visit is for medication renewals or when the patient has additional health concerns that need attention from their physician in the same visit. There will be four key components of the PT-led primary care intervention: 1) initial assessment and screening; 2) brief individualized intervention at the first visit; 3) health services navigation; 4) providing additional PT care for people with an unmet need. For the pilot study, we will have one PT providing care for both of the primary care practices in the PT-led primary care model arm.

All participants who see the PT in primary care will be able to book follow-up appointments. However, to avoid duplication of available PT services, only those with an identified need and no financial coverage for physiotherapy services (i.e., no health insurance coverage for PT and not meeting the criteria for PT funded by the ministry of health in Ontario) will be scheduled for a follow-up appointment with the PT in primary care at the end of the initial visit.

#### Assessment and screening

**Fig. 1:**
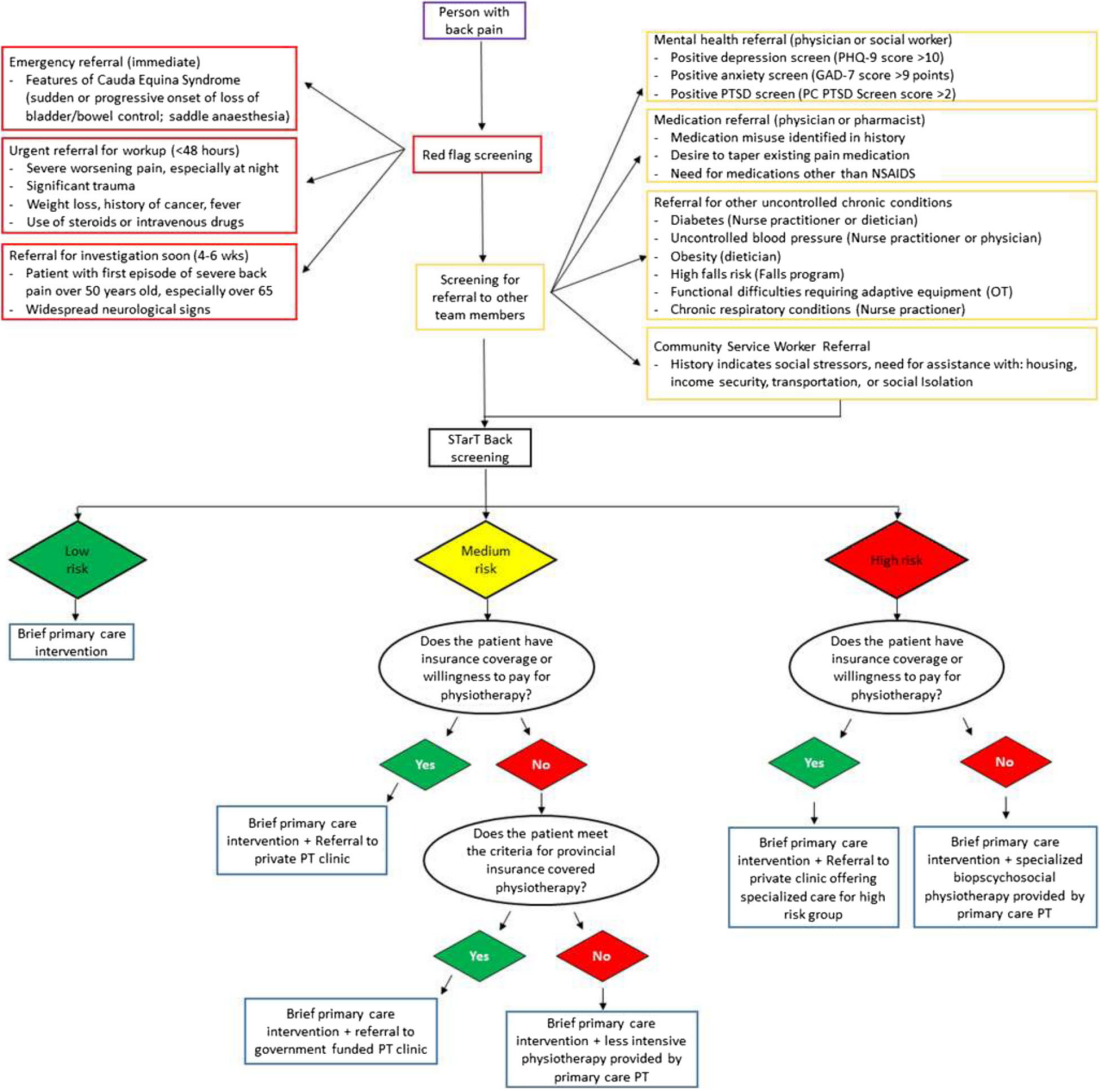
Overview of the PT in primary care screening and navigation process. The physiotherapist (*PT*) will help the patient navigate the healthcare system in three stages. First, any red flags identified will lead to emergency, urgent, or soon referrals to the emergency department, physician, and/or imaging. Second, the patient history, examination findings, and validated questionnaires, when appropriate, will be used to determine if other healthcare providers are needed for this patient’s care. Third, the PT will use the STarT back risk stratification tool (individuals screened as low, medium, or high risk of ongoing pain and disability [27]) to guide referral pathways for physiotherapy. The matched low-risk pathway is a brief primary care intervention (reassurance, advice, and exercise) with no referral. The medium-risk pathway involves a referral for usual physiotherapy care in addition to the brief intervention. The high-risk matched pathway includes referral for a combined physical and psychologically informed treatment approach which aims to address barriers to recovery, facilitate increases in activity, and address unhelpful back pain beliefs and behaviors. This treatment approach has been described in more detail elsewhere [56]. *GAD-7* General Anxiety Disorder-7, *NSAIDS* nonsteroidal anti-inflammatory drugs, *OT* occupational therapy, *PC* primary care, *PHQ-9* Patient Health Questionnaire-9, *PTSD* post-traumatic stress disorder

The assessment and screening will include: taking a history; screening for red flags (e.g., signs/symptoms associated with cauda equina syndrome, traumatic fracture, cancer, or widespread neurological symptoms); physical and neurological examination; application of evidence-based tools to identify comorbid health conditions (e.g., depression) that require additional care [46, 47]; and using a validated tool (STarT Back [27, 28]) to identify physical and psychosocial risk factors associated with ongoing pain and disability (Fig. 1).

**Fig. 2:**
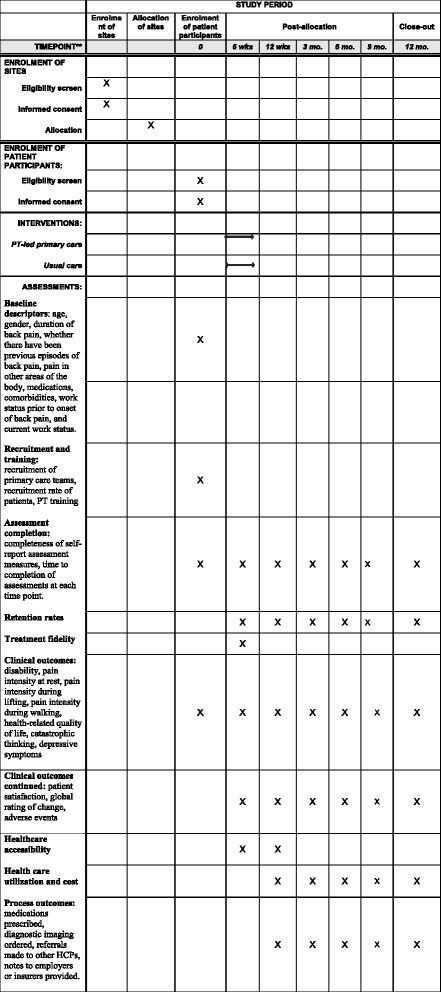
Schedule of enrolment, interventions, and assessments

#### Brief individualized intervention

The PTs will provide a brief individualized intervention at the initial visit. This intervention will be based on primary care guidelines for back pain [22] and will consist of effective communication to validate the patient’s experiences [48] and allow the patient to disclose the impact of their back pain on their lives [49, 50], as well as providing cognitive reassurance [51], a few exercises [52, 53], and advice/strategies to stay active [54]. In line with evidence for effective education, the recommendations will be supported with written information [55].

#### Health services navigation

The PT will assist the patient with back pain in navigating the appropriate healthcare services based on the assessment findings (Fig. 1). First, they will identify any red flags requiring emergency or urgent referrals. Next, they will identify any comorbid conditions that need attention from other members of the healthcare team; for example, people who screen positive for depression will be referred to their physician or a member of the mental healthcare team. Finally, they will make a referral to PT (if appropriate) informed by the patient’s score on the STarT Back tool [27, 28]. The STarT Back tool effectively categorizes patients with back pain into low, medium, or high risk of ongoing pain and disability based on physical and psychosocial risk factors for poor recovery [27]. Low-risk patients will receive only the brief individualized intervention at intake, medium-risk patients will receive a referral for standard community physiotherapy, and the high-risk patients will be referred to a PT with specific training in combined physical and psychologically informed practice aimed at reducing risk factors for chronic pain and disability [56, 57]. The PT in the primary care team will refer people classified as medium or high risk to a community PT based on the financial resources available to the patient (e.g., private PT referral when the patient has private health insurance for PT or government-funded physiotherapy for those who meet the specified criteria).

The STarT Back stratified approach to back pain management has been shown in a UK study to improve function, quality of life, and cost-effectiveness in comparison to usual care [28]. However, this stratified approach has been difficult to implement in contexts outside of the UK due to differences in funding models for physiotherapy. For example, people classified as low risk who have extended health insurance often use their coverage for PT care, even though the STarT Back approach suggests these individuals are likely to experience a full recovery without PT intervention. The PT-led primary care model for back pain utilized in this study will aim to overcome this barrier using education and shared decision making with patients classified as low risk. The education will help patients understand that they are likely to recover without additional PT services so that they can make an informed decision about whether or not to seek additional care using this knowledge. Another barrier to using the STarT Back stratified approach to care in the Canadian context is that people classified as medium or high risk often do not have financial coverage for PT. The PT-led primary care model for back pain utilized in this study will address this barrier by providing ongoing PT care for people identified with an unmet need.

#### Providing additional physiotherapy care to people with an unmet need

The PT in primary care will provide additional physiotherapy care (at the primary care site) to patients who are classified as medium and high risk of ongoing pain and disability (based on STarT Back screening) and who do not have access to physiotherapy coverage through either private or government health insurance plans. The care will include evidence-based strategies, such as individualized education [58], exercise [52, 53], and cognitive behavioral approaches [56].

### Physiotherapist training

The registered physiotherapist involved in the new PT-led primary care model for back pain will participate in 5 days of training including:

i. a review of red-flag screening;
ii. screening for comorbidities and services available within the participating family health teams and community health center for people with these conditions;
iii. examination of back pain including subjective and physical assessment according to clinical practice guidelines;
iv. using and interpreting outcome measures and screening tools for people with back pain;
v. diagnostic imaging guidelines for back pain and how to appropriately utilize imaging findings from a radiologist in clinical decision making;
vi. using the STarT Back tool to classify people with back pain into low, medium, or high risk of ongoing pain and disability, and how to refer to a PT based on the classification;
vii. PT services available in the Kingston, Ontario, area based on patient healthcare resources (e.g., private clinics, Ontario Health Insurance Plan-funded physiotherapy clinics)
viii. the brief primary care intervention for people with back pain, including cognitive reassurance, education regarding prognosis and the importance of continued participation in usual life-role activities, and brief prescription of physical activity and exercise;
ix. activity-based PT for people identified at medium risk of ongoing pain and disability based on STarT Back screening. This will include education, graded activity, and exercise.
x. additional interventions aimed at reducing psychosocial risk factors for people classified at high risk of ongoing pain and disability by the STarT back screening. This will include communication skills to facilitate personal disclosure, pain neurophysiology education, de-catastrophizing interventions, graded exposure to reduce activity-related fear, pacing strategies, cognitive-behavioral strategies to improve self-efficacy and facilitate behavior change, as well as strategies to improve sleep, manage stress, and manage flare-ups

### Usual physician-led care model

The physician-led primary care intervention will be unstandardized to best reflect standard clinical practice in Canada. This usually includes a visit to a primary care physician, who would perform a history and physical examination, provide back pain education, and prescribe medications and/or refer based on their assessment findings and patient preferences. Participants in both groups will be permitted to seek additional care which will be monitored through a healthcare utilization questionnaire administered at all follow-up assessments.

### Evaluation and outcomes

The feasibility outcomes described below will be the primary outcomes of this pilot study. These measures will be used to inform a full-scale trial including how many sites to involve, which measures to include, and how to effectively train the PTs. Secondary outcomes will include the clinical, health system, and process outcomes planned for the full trial. We will determine the feasibility for collecting these outcomes and report them descriptively, but no comparisons will be made between groups. Data collection forms are available from the authors upon request.

#### Feasibility outcomes

##### Recruitment of primary care teams

Recruitment and retention of four family health teams or community health centers will be an essential feasibility criterion for proceeding with the full trial.

##### Recruitment of patients

The feasibility of patient recruitment will be determined by the overall recruitment rate. The full trial will be considered feasible if, during the pilot study, we are able to recruit 21 patients over 13 weeks using four sites. This recruitment rate will allow us to exceed our preliminary sample size calculated for the full trial (*n* = 640) with 16 sites over 2.5 years. This sample size will be recalculated based on the pilot data, and the recruitment rate from this pilot study will inform whether our preliminary plan of using 16 sites over 2.5 years is feasible or if additional sites or a longer recruitment period are needed.

##### Assessment procedures

Feasibility of the assessment procedures will be measured by completeness of data and duration of completing all outcome measures among all people that start each assessment. As recommended for pilot studies, we set criteria a priori for acceptable completeness [59], and will consider > 80% of all assessment items completed and a mean time for completion of < 60 min as acceptable.

##### Retention

Retention will be assessed by attrition rate, with < 20% attrition at 12-month follow-up considered indicative of a fully successful pilot with a plan of using the retention strategies from the pilot in the full trial, < 35% attrition will be considered partially successful with a plan to proceed with the full trial, but with additional retention strategies identified and implemented for the full trial based on evidence that > 20% attrition threatens trial validity [60].

##### Physiotherapist training

Feasibility of training the PT who will adopt the primary care role will be evaluated through attendance, ratings of self-efficacy (0–10) for delivering each of the four components of the intervention after training, and qualitative feedback. A successful outcome will be considered as full attendance in the training and self-efficacy ratings of at least 8/10 on each component of the intervention. Qualitative feedback will be used to inform the PT training in the full trial.

##### Physiotherapist treatment fidelity

Treatment fidelity will be encouraged through training and by providing a treatment fidelity checklist to the participating PTs. Fidelity will be measured through an audit of the fidelity checklist and electronic medical record (EMR) of each included patient to determine consistency of the intervention with the protocol [61]. An acceptable level of fidelity will be considered > 80% for red-flag screening, reassurance, advice to stay active, exercises, and referral consistent with the intervention decision tree.

#### Baseline factors used to describe the population

In order to describe the population at baseline, we will collect the following baseline information from participants: age, gender, duration of back pain, whether there have been previous episodes of back pain, whether or not the participant has pain in other areas of the body, medications, comorbidities, work status prior to onset of back pain, and current work status.

#### Clinical outcomes

In preparation for the future fully powered trial, we will pilot the data collection for the clinical outcomes via this pilot study. All measures will be collected through either electronic or paper data collection forms with the assistance of a trained research assistant at baseline, 6 weeks, and 3, 6, 9, and 12 months, with the primary time point for comparison being the 12-month follow-up (Fig. 2). The following clinical effectiveness outcomes will be piloted in this study.

Self-reported disability will be made using the Roland Morris Questionnaire (RMQ), which demonstrates reliability, validity, and responsiveness in people with acute and chronic back pain [62, 63].

Pain intensity will be measured using a Numeric Pain Rating Scale (NPRS) at sitting, which has shown good reliability and responsiveness in acute and chronic back pain [64]. We will ask patients to report their pain at rest and during two physical activities (walking and lifting a grocery bag) based on evidence that exercise-evoked pain may respond differently to treatment than pain at rest [65–67].

Health-related quality of life will be reported using the EuroQOL-5D (EQ-5D) [68], a preference-based measure that performs well in LBP and is suitable for economic evaluation in this population [69]. The EQ-5D score will also be converted to quality-adjusted life years (QALY) using the UK VAS A3 value set as recommended in the absence of Canadian value sets [68].

Global rating of change will be assessed using an 11-point global rating of change scale (GROC) (–5 to +5) as has been recommended in the literature for self-reported rating of change [70, 71].

Patient satisfaction will be assessed using an 11-point scale with anchors of very dissatisfied (–5) and very satisfied (+5).

Catastrophic thinkin*g* will be assessed using the 13-item Pain Catastrophizing Scale (PCS) [72].

Depressive symptoms will be assessed using the nine-item Patient Health Questionnaire (PHQ-9) [73].

Adverse events will be reported using an adverse events questionnaire that is reported to be consistent with reporting guidelines [74, 75], and asks: 1) if the patient has experienced any events as a result of any of the treatments received (yes/no); 2) how long the event lasted (hours or days); 3) how severe the adverse event was (0–10 scale); and 4) what adverse events were experienced. Any serious adverse events will be addressed immediately by referral to the most appropriate member of the primary healthcare team.

#### Health system outcomes

We will pilot data collection needed to analyze the following health system outcomes:

Accessibility will be measured using the percentage of patients with back pain who are assessed by a primary care provider within 48 h and the percentage of patients who score medium or high risk on the STarT Back screening tool who access physiotherapy (as endorsed by guidelines [76]).

Healthcare utilization will be assessed using a self-report questionnaire at each assessment focused on the following health services: primary care visits (physicians, nurse practitioners, PT in primary care), emergency room visits, hospitalizations, surgeries, consultations with other healthcare professionals (e.g., other PTs, chiropractors, physician specialists, clinics), diagnostic imaging (e.g., x-ray, computed tomography (CT), magnetic resonance imaging (MRI)), medications, and other costs borne by the participant (e.g., other treatment).

#### Cost outcomes

We will pilot data collection to be able to perform a cost utility analysis in the full trial.

##### Sources of direct healthcare cost data

Intervention costs will include the PT salary, training, materials, and space needed to carry out the intervention. Costs for publically funded healthcare services will be calculated using the Ontario Ministry of Health and Long-term Care Schedule of Benefits [77]. Medication costs will be obtained from the Ontario Drug Benefit formulary. For private healthcare services, the mean cost for the services in the community served will be used (e.g., we surveyed PTs in Kingston and found mean costs of $80 for initial and $60 for follow-up visits).

##### Indirect costs

Non-healthcare costs will be limited to loss of productivity (LOP) using a human capital approach. The mean Canadian wage reported by Statistics Canada will be used to assign a monetary value to time lost from paid employment by both patients and caregivers collected via self-report measures at each follow-up assessment. The minimum wage value in Ontario will be used to place a value on time lost by those who were retired as well as on time away from volunteer or homemaking activities.

#### Process outcomes

We will pilot the collection of process outcomes to determine the feasibility of comparing differences in management between groups in the full trial. The following process outcomes will be collected from the electronic medical record: medications prescribed, diagnostic imaging ordered, referrals made to other HCPs (both internal and external to the primary healthcare team), the number of visits to members of the primary care team, and notes to employers or insurers provided. Number of visits to HCPs outside of the primary care team will be recorded via patient diary.

### Data collection and management

All data will be collected on electronic or paper data collection forms (depending on participant preference). Participants will be instructed on how to complete the questionnaires by trained research assistants and data will be entered into an encrypted and secure study database. Personal identifying information including name, date of birth, and contact information will be collected for obtaining data from the electronic medical record and for contacting participants for follow-up appointments. This personal information will be stored in a separate file from participant data that is also encrypted and password protect. The file that contains personal identifiers will be destroyed at the end of the trial and de-identified data will be permanently anonymized. Only study investigators and research staff will have access to the data. Data quality will be assured by checking 10% of data from the database with original data collection forms. Research assistants will schedule follow-up visits with participants at the initial visit and will call or email (based on participant preference) 1 week in advance to remind the participant of their approaching assessment. Multiple attempts will be made to contact participants by phone or email for any participants who miss an appointment without cancellation.

### Analysis

In keeping with methodological recommendations and reporting guidelines for pilot and feasibility studies [59, 78, 79], all feasibility outcomes will be reported descriptively and analyzed qualitatively. Descriptive statistics for each of the clinical, health system, and process outcomes will be reported in aggregate only to avoid potentially influencing a decision about proceeding with the full trial based on preliminary data. We will use means (standard deviation) for continuous variables that are normally distributed or medians (interquartile range) for continuous variables that are not normally distributed. Categorical variables will be presented as count and percentage for each category. The total costs will be determined by multiplying the quantity of resource use by the corresponding unit cost, summing the total cost over each follow-up interval, and then calculating the mean cost at each follow-up time point, as well as an overall mean cost for the entire study period. Results will be presented as aggregated and disaggregated costs. We will document challenges in collection of economic data to inform the plan for the full trial, where we intend to perform a complete cost-utility analysis. We will perform an interim analysis after the recruitment period, 6-week, and 3-month follow-ups are completed for all participants in order to plan for the full trial. The remaining follow-ups (6, 9, and 12 months) will be analyzed after the final participant has completed the 12-month follow-up.

### Sample size

The preliminary sample size calculated for the planned fully powered cluster randomized trial is 16 practices with an average of 40 patients per practice (640 patients in total). This will achieve 90% power to detect a minimally important mean difference of 2.5 points on the RMQ [80] at 3-month follow-up (Cohen’s *d* = 0.4) using a two-sided α = 0.05 and assuming a standard deviation of 6.2 points [28], an intracluster correlation coefficient of 0.01 [81], an expected variation in recruitment per practice using a coefficient of variation of 0.65 [82], and accounting for 20% attrition. This calculation is preliminary and we will re-visit this sample size calculation based on the data obtained from this pilot study. For this pilot study, we plan to recruit patients from four primary care teams over a period of 14 weeks. The pilot sample size is based on logistical considerations rather than formal power considerations. The four primary care sites that will be invited to participate have approximately 24 full-time equivalent (FTE) physicians in total, averaging 26 clinical hours/week, a minimum of two patients/clinical hour, and an anticipated 1.5% of visits for back pain. Based on 25% enrolment in our previous work [28], we anticipate 65 patients over the 14-week recruitment period, far exceeding our feasibility target of 21 patients over 13 weeks.

### Associated qualitative interviews and focus groups

To explore the perspectives of patients, we will interview approximately 10 to 15 patients who received care at the sites that incorporated a PT in the primary care team. We will use purposive sampling to identify participants who have been classified as low, medium, and high risk based on the STarT Back classification. Furthermore, we will sample from participants who are appropriate for additional PT care who have received a referral and those that have received ongoing care from the PT in the primary care setting. We will continue with interviews until saturation. We will use semi-structured interview guides that explore experiences and attitudes towards the new service delivery model, barriers/facilitators to implementation, perceived satisfaction, perceived value, and impact on clinic processes and patient outcome. The data will be recorded, transcribed verbatim, and coded in an interpretive description tradition [83, 84] to develop clinically meaningful themes. Two investigators will independently review interview transcripts and identify themes using interpretive description [83, 84]. The findings from these qualitative interviews will provide rich data upon which to inform any necessary changes for the full trial, including changes to the PT training, implementation of the intervention and assessment procedures, and communication between the researchers and healthcare team.

To explore the perspective of HCPs, we will conduct two focus groups with each of the primary care teams who integrate a PT within their team. Similar to the interviews with patients, the focus groups will ask HCPs about their experiences and attitudes towards the new service delivery model, barriers/facilitators to implementation, perceived satisfaction, perceived value, and impact on clinic processes and patient outcomes.

### Protocol amendments

Protocol amendments will be will be communicated by updating the trial registry at ClinicalTrials.gov, contacting all investigators and participants, and reporting in the final trial publication.

### Knowledge translation and dissemination

This pilot study will serve as an important opportunity to engage target stakeholders (patients, HCPs, administrators, policy makers) who will support effective knowledge translation (KT) activities throughout the full trial. During the pilot study we will solicit feedback on the protocol from members of each of these target stakeholder groups to inform the conduct of the fully powered trial and will work with these stakeholders to develop a clearly articulated KT plan for the full trial. Our team knowledge users (LC and CM) will work with investigators to develop the KT plan. Findings from this pilot study will be presented at local rounds at participating sites, provincial and national scientific conferences, and published in journals in the fields of primary care or rehabilitation. Authorship will be determined using the International Committee of Medical Journal Editors (ICMJE) recommended authorship criteria.

## Discussion

This study will determine the feasibility of a cluster randomized controlled trial investigating the impact of a new PT-led primary care model for back pain. If this pilot demonstrates feasibility, a fully powered trial will provide evidence that has the potential to transform primary care for back pain in Canada. The full trial will inform future service design and whether these models should be more widely implemented. It will also inform workforce development and training agendas. Expansion of the primary care team is being advocated as one way of meeting increasing demand and, although this study focuses on back pain and PTs, the findings will have relevance to other clinical conditions and healthcare professionals.

## Additional files

Additional file 1: Informed consent form for study participants. (DOCX 55 kb)

Additional file 2: SPIRIT Checklist. (DOC 121 kb)
