# Supplementary material for: Determining the impact of a new physiotherapist-led primary care model for back pain: protocol for a pilot cluster randomized controlled trial
Source: Trials. 2017 Nov 9;18:526. doi: 10.1186/s13063-017-2279-7 (PMC5680754; doi:10.1186/s13063-017-2279-7)
Supplement: Supplementary file 1 — Informed consent form for study participants. (DOCX 55 kb) [file 13063_2017_2279_MOESM1_ESM.docx]

Additional file 1

Informed Consent Form for Participation in a Research Study

**Study Title**: Determining the impact of a new physiotherapist led primary care model

for low back pain – A pilot cluster randomized controlled trial

**Study Principal Investigator**:

Jordan Miller, Assistant Professor, School of Rehabilitation Therapy, Queen’s University

Phone: 905-872-9938

Email: [jordan.miller@queensu.ca](mailto:jordan.miller@queensu.ca)

**Study Co-investigators:** David Barber, Catherine Donnelly, Simon French, Michael Green, Jonathan Hill, Joy MacDermid, Jacquelyn Marsh, Kathleen Norman, Julie Richardson, Monica Taljaard, Timothy Wideman

**Study Sponsor:** Canadian Institutes of Health Research (#384307)

INTRODUCTION

You are being invited to participate in a clinical trial (a type of study that involves research). You are invited to participate in this trial because you have back pain. This consent form provides you with information to help you make an informed choice. Please read this document carefully and ask any questions you may have. All your questions should be answered to your satisfaction before you decide whether to participate in this research study.

Taking part in this study is voluntary. Deciding not to take part or deciding to leave the study later will not result in any penalty or affect current or future health care.

WHAT IS THE BACKGROUND INFORMATION FOR THIS STUDY?

Most often, people with back pain who are looking for health care for their back pain go to see their family doctor. The doctor then performs an assessment and makes treatment recommendations that might include advice, medication, or a referral to another health care provider such as a physiotherapist. Other people go to see a physiotherapist for their first visit, where the physiotherapists performs an initial assessment and makes treatment recommendations based on their assessment findings. These treatments might include advice, exercise, or referral to another health care provider. It is not clear whether there is a difference in results in people who see a family doctor first for their back pain or those who see a physiotherapist first for their back pain.

WHY IS THIS STUDY BEING DONE?

The purpose of this study called a pilot study, is to test the study plan and to find out whether enough participants will join a larger study and accept the study procedures. This type of study involves a small number of participants and so it is not expected to prove whether seeing a physiotherapist first or seeing a physician first results in better outcomes. The results may be used as a guide for larger studies, although there is no guarantee that they will be conducted. Participation in a pilot study does not mean that you will be eligible to participate in a future larger study.

WHAT OTHER CHOICES ARE THERE?

You do not have to take part in this study in order to receive treatment or care. You can take part in other treatment options regardless of whether you take part in this study. Additional therapies that you could discuss with your doctor or physiotherapist include: medication, physiotherapy, chiropractic care, or no therapy.

HOW MANY PEOPLE WILL TAKE PART IN THIS STUDY?

Because this is a pilot study, we don’t know exactly how many people will take part. It is anticipated that between 20 and 100 people will take part in this study from four different primary care teams in the Kingston area over a 4-month period. This study should take 12 months to complete and the results should be known in 18 months.

WHAT WILL HAPPEN DURING THIS STUDY?

In this study, your health team has been "randomized" into either having a physiotherapist available to patients for their first visit or not having a physiotherapist available for their first visit. Randomization means that your health care site was put into a group by chance (like flipping a coin). Your health care will not be influenced by whether or not you agree to participate in this study. If you are at a site without a physiotherapist, you will see your family doctor for your visit. If you are at a site with a physiotherapist, you will be given the opportunity to see a physiotherapist or your family doctor. Whether or not you participate in this study will not influence this choice. You will have the opportunity to choose to see either the physiotherapist or family doctor if you are at one of these sites.

WHAT IS THE STUDY INTERVENTION?

Group 1: Sites without a physiotherapist. At these sites, you will see your family doctor or another member of your health care team. This is “usual care” because this is what often happens at your health care team.

Group 2: Sites with a physiotherapist. At these sites, you will have the choice of seeing a physiotherapist for your initial appointment or seeing your family doctor. Your health care team encourages you to see the physiotherapist for your back pain unless you have multiple health concerns to address with your appointment or are booking the appointment with the purpose of just renewing your medications.

In either group, you will take part in an assessment of your back pain and treatment options will be discussed for you to decide upon with your doctor or physiotherapist. These could include: advice, medications, exercises, or a referral to another health care provider.

WHAT ARE THE STUDY PROCEDURES?

Questionnaires

You will be asked to complete a series of questionnaires as part of this study. Some of these questionnaires may be done as part of your standard care, in which case the results may be used. Some of these tests may be done more frequently than if you were not taking part in this study and some may be done only for the purpose of the study. The questionnaires include questions about your:

- Age
- Gender
- Other health conditions you have
- Medications you are taking
- Your work or employment
- Health care you have received for your back pain
- The costs you have had as a result of your back pain
- How long you have been having back pain
- How intense your back pain is
- How the back pain has been influencing your day-to-day activities
- Your thoughts, feelings, and emotions related to your back pain

The questionnaires will take about 30 minutes to complete. You will be asked to complete these questionnaires at your first visit, after 6-weeks, 3-months, 6-months, 9-months, and 12 months.

The information you provide is for research purposes only. Some of the questions are personal. You can choose not to answer questions if you wish.

Even though you may have provided information on a questionnaire, these responses may not be reviewed by your health care team. -If you wish them to know this information please bring it to their attention.

Access to your medical records

Some information will be collected from your electronic medical record as part of this study. The purpose of this information is to determine whether the health care provided differs between sites who have a physiotherapist and sites that do not, as well as determining whether there is a difference in the health care used. The information collected from your electronic medical record will include: health care visits with any members of the health care team, medications prescribed, referrals for other treatment (e.g. physiotherapist, chiropractor, specialist physician) or imaging (e.g. xrays), and whether any notes were provided to employers or insurers.

Optional Interviews

The Researchers doing this study are interested in understanding the experience of people who receive care from both doctors and physiotherapists. After your 6-week assessment, you will be asked to participate in an optional interview with a research staff member. You will be given an additional optional study consent form to read and sign if you wish to give permission for this. You may decide not to participate in the optional research and still participate in this main study.

WHAT ARE THE RESPONSIBILITIES OF STUDY PARTICIPANTS?

If you choose to participate in this study, you will be expected to:

- Tell the research staff and health care providers about your current medical conditions and other treatments you are receiving;
- Return any questionnaires that you take home to complete or that you ask to receive by email or phone

HOW LONG WILL PARTICIPANTS BE IN THE STUDY?

You will be asked to come back to the health center (your family doctors office) after 6-weeks, 3-months, 6-months, 9-months, or 12-months. If you are unable to attend these appointments, a research assistant can contact you by email or phone so that you can fill out the questionnaires online or by phone.

No matter which group your health team are randomized to, we would like to keep track of your health for 12 months. We would do this by asking you to complete the questionnaires at all follow-up time points regardless of which group you are a part of.

CAN PARTICIPANTS CHOOSE TO LEAVE THE STUDY?

You can choose to end your participation in this research (called withdrawal) at any time without having to provide a reason. If you choose to withdraw from the study, you are encouraged to contact the study staff.

You may withdraw your permission to use information that was collected about you for this study at any time by letting the study staff know. However, this would also mean that you withdraw from the study.

WHAT ARE THE RISKS OR HARMS OF PARTICIPATING IN THIS STUDY?

Participating in this study does not directly influence the care provided by your family doctor or physiotherapist; however, differences in practice patterns between physiotherapists and physicians may still indirectly influence your risk of some side effects. For example, physiotherapists may be more likely to give you some exercises for your back pain. These exercises may produce short-term increases in soreness. If you experience any side effects, you should report them to your doctor or physiotherapist.

WHAT ARE THE BENEFITS OF PARTICIPATING IN THIS STUDY?

There are no direct medical benefits to you for taking part in this study. If your family doctor is at a site with a physiotherapist, you may have access to physiotherapist services when you otherwise may not have had access. These services will be available regardless of your participation in the study and whether or not you drop out.

We hope the information learned from this study will help other people with back pain in the future.

HOW WILL PARTICIPANT INFORMATION BE KEPT CONFIDENTIAL?

If you decide to participate in this study, the study doctors and study staff will only collect the information they need for this study.

Records identifying you will be kept confidential and, to the extent permitted by the applicable laws, will not be disclosed or made publicly available, except as described in this consent document.

Authorized representatives of the Queen's University Health Sciences and Affiliated Teaching Hospitals Research Ethics Boardmay look at your original (identifiable) medical/clinical study records at the site where these records are held, to check that the information collected for the study is correct and follows proper laws and guidelines.

This study requires the transfer of your study data to Queen’s University for the purposes of analyzing the data. The information you provide will be stored in a secure online database and accessed on password protected computers at Queen’s University. The information you provide and that comes from the medical record will be coded and not associated with your name or any other personal identifiers when it is transferred.

If the results of this study are published, your identity will remain confidential. It is expected that the information collected during this study will be published in scientific journals and presented at conferences. Even though the likelihood that someone may identify you from the study data is very small, it can never be completely eliminated.

A copy of the consent form that you sign to enter the study may be included in your health record/hospital chart.

WHAT IS THE COST TO PARTICIPANTS?

There are no costs to you to participate. The costs of your medical treatment will be paid for by your provincial medical plan to the extent that such coverage is available. The physiotherapist salary for sites with the physiotherapist is funded through the research sponsor.

If you are at a site with the physiotherapist, you may not be able to receive ongoing care from the physiotherapist after your participation in the study is completed. The physiotherapist’s salary is covered by research funds and after the study is completed, you may decide it is too expensive and insurance coverage may not be available.

ARE STUDY PARTICIPANTS PAID TO BE IN THIS STUDY?

You will not be paid for taking part in this study. If you decide to participate in this study, you will receive $20 for each visit to cover any costs incurred, such as transportation or parking.

In the case of research-related side effects or injury, medical care will be provided by your health team.

WHAT ARE THE RIGHTS OF PARTICIPANTS IN A RESEARCH STUDY?

You will be told, in a timely manner, about new information that may be relevant to your willingness to stay in this study.

You have the right to be informed of the results of this study once the entire study is complete. If you would like to be informed of the results of this study, please let the research staff know.

Your rights to privacy are legally protected by federal and provincial laws that require safeguards to ensure that your privacy is respected.

By signing this form you do not give up any of your legal rights against the study doctor or involved institutions for compensation, nor does this form relieve the study doctor or their agents of their legal and professional responsibilities.

You will be given a copy of this signed and dated consent form prior to participating in this study.

WHOM DO PARTICIPANTS CONTACT FOR QUESTIONS?

If you have questions about taking part in this study, or if you suffer a research-related injury, you can talk to the research staff or the researcher who is in charge of the study at this institution.

Research coordinator:

Kevin Varette

Phone: 613-888-5943

Email: varettek@queensu.ca

Principal investigator:

Jordan Miller

Phone: 905-872-9938

Email: jordan.miller@queensu.ca

If you have any questions about your rights as a participant or ethics concerns please contact the Queen’s University Health Sciences and Affiliated Teaching Hospitals Research Ethics Board (HSREB) at 1-844-535-2988 (Toll free in North America) or email the HSREB Chair at clarkaf@queensu.ca.

SIGNATURES

- All of my questions have been answered,
- I understand the information within this informed consent form,
- I allow access to my medical records as explained in this consent form,
- I do not give up any of my legal rights by signing this consent form,
- I agree to take part in this study.

____________________________ ______________________ _________________

Signature of Participant PRINTED NAME Date

____________________________ ______________________ _________________

Signature of Person Conducting PRINTED NAME & ROLE Date

the Consent Discussion
